# Causal associations between thyroid dysfunction and COVID-19 susceptibility and severity: A bidirectional Mendelian randomization study

**DOI:** 10.3389/fendo.2022.961717

**Published:** 2022-09-06

**Authors:** Zhihao Zhang, Tian Fang, Yonggang Lv

**Affiliations:** ^1^ Department of Thyroid Breast Surgery, Xi’an NO.3 Hospital, the Affiliated Hospital of Northwest University, Xi'an, China; ^2^ Department of Medical Oncology, Cancer Center, West China Hospital of Sichuan University, Chengdu, China

**Keywords:** COVID-19, hypothyroidism, hyperthyroidism, Mendelian randomization, thyroid dysfunction

## Abstract

**Background:**

Observational studies have reported an association between coronavirus disease 2019 (COVID-19) risk and thyroid dysfunction, but without a clear causal relationship. We attempted to evaluate the association between thyroid function and COVID-19 risk using a bidirectional two-sample Mendelian randomization (MR) analysis.

**Methods:**

Summary statistics on the characteristics of thyroid dysfunction (hypothyroidism and hyperthyroidism) were obtained from the ThyroidOmics Consortium. Genome-wide association study statistics for COVID-19 susceptibility and its severity were obtained from the COVID-19 Host Genetics Initiative, and severity phenotypes included hospitalization and very severe disease in COVID-19 participants. The inverse variance-weighted (IVW) method was used as the primary analysis method, supplemented by the weighted-median (WM), MR-Egger, and MR-PRESSO methods. Results were adjusted for Bonferroni correction thresholds.

**Results:**

The forward MR estimates show no effect of thyroid dysfunction on COVID-19 susceptibility and severity. The reverse MR found that COVID-19 susceptibility was the suggestive risk factor for hypothyroidism (IVW: OR = 1.577, 95% CI = 1.065–2.333, *P* = 0.022; WM: OR = 1.527, 95% CI = 1.042–2.240, *P* = 0.029), and there was lightly association between COVID-19 hospitalized and hypothyroidism (IVW: OR = 1.151, 95% CI = 1.004–1.319, *P* = 0.042; WM: OR = 1.197, 95% CI = 1.023-1.401, *P* = 0.023). There was no evidence supporting the association between any phenotype of COVID-19 and hyperthyroidism.

**Conclusion:**

Our results identified that COVID-19 might be the potential risk factor for hypothyroidism. Therefore, patients infected with SARS-CoV-2 should strengthen the monitoring of thyroid function.

## Introduction

Coronavirus disease 2019 (COVID-19) is an infectious disease caused by infection with severe acute respiratory syndrome coronavirus 2 (SARS-CoV-2). It has rapidly become a global epidemic and a serious health threat (1). Currently, several studies have shown that some susceptible populations include type 2 diabetes, obesity, hypertension, and other metabolic diseases (2–5). However, thyroid disease is not covered in the report.

SARS-CoV-2 virus infection and thyroid-promoted inflammatory responses are related and interact in a complex way. Several small observational case studies now support that thyroid dysfunction is a predisposing factor for COVID-19 (6, 7) and that hospital mortality is higher in patients with COVID-19 hypothyroidism than in patients with COVID-19 normal thyroid function (8). However, some studies have taken the opposite view (9–11), suggesting that the real cause of COVID-19 progression is antithyroid drug-induced neutropenia rather than thyroid disease status. In addition, SARS-CoV-2 may impair the hypothalamic–pituitary–thyroid axis or damage thyroid problems through a viral inflammatory process, which may increase the risk of future partial thyroid dysfunction (12–14). However, these findings remain vulnerable to confounding factors and reverse causality that cannot be fully excluded in observational studies. Further investigation is needed to determine the causal relationship between thyroid dysfunction and COVID-19 infection.

Mendelian randomization (MR) is a new epidemiological approach that uses genetics as a tool to study correlations between exposure and outcome (15). MR is biologically based on the random distribution of gametes in meiosis and has the advantage of overcoming the limitations of confusion and reverse causality often encountered in observational studies (16). In this study, we performed bidirectional MR to explore the causal association between thyroid dysfunction and COVID-19 risk.

## Methods

### Study design

We conducted a bidirectional two-sample MR study to assess the causal relationship between thyroid dysfunction (hypothyroidism and hyperthyroidism) and COVID-19 susceptibility and its severity. The instrumental variables must satisfy three basic principles: (1) Genetic variation is strongly correlated with exposure; (2) genetic variation is not strongly related to potential confounders; and (3) genetic variation does not directly affect the outcome (17). We performed bidirectional MR in this study, and forward MR: thyroid dysfunction genome-wide association study (GWAS) data were used to explore the effect of thyroid dysfunction on COVID-19. Reverse MR: COVID-19 GWAS was used as an exposure to explore the effect of COVID-19 on thyroid dysfunction (**Supplementary Figure 1**).

### Data sources

Data from genome-wide association studies of COVID-19 cases were obtained from the COVID19 Host Genetics Initiative GWAS meta-analysis, round 5 (18). In our study, the COVID-19 susceptibility phenotype comprised 38,984 European patients with COVID-19 defined as individuals with laboratory confirmation of SARS-CoV-2 infection or electronic health records [using the International Classification of Diseases (ICD) or physician annotations] or self-reported, with 1,644,784 control individuals. We then used two cohorts to assess the COVID-19 severity phenotype. The first cohort was compared to 9,986 hospitalized patients versus 1,877,672 control individuals. The second cohort compared 5,101 very severe patients, defined as patients who died or required respiratory support (including continuous positive airway pressure (CPAP), bilevel positive airway pressure (BiPAP), intubation, or high-flow nasal cannula). A total of 1,383,241 control individuals were not included as cases (https://www.COVID19hg.org/blog/2021-03-02-freeze-5-results/). Details of the phenotypes are shown in **Table 1**.

**Table 1:** Sources of data for the analysis.

Phenotype	Source of Genetic Variants	
	Consortium	Participants
**Hypothyroidism**	–	**Cases:** 3,340 cases with TSH levels above the reference range. **Controls:** 49,983 individuals with TSH levels in the reference range.
**Hyperthyroidism**	–	**Cases:** 1,840 cases with TSH levels below the reference range. **Controls:** 49,983 individuals with TSH levels in the reference range.
**COVID-19 susceptibility**	**Susceptibility**	**Cases:** 3,8984 individuals with COVID-19 by laboratory confirmation of SARS-CoV-2 infection, or by electrical health records (using ICD or physician notes), or self-reporting. **Controls:** 1,644,784 individuals enrolled in the cohorts and not included as cases.
**COVID-19 severity**	**Hospitalized**	**Cases:** 9,986 hospitalized individuals with COVID-19. **Controls:** 1,877,672 individuals enrolled in the cohorts and not included as cases
	**Very severe disease**	**Cases**: 5,101 very severe patients defined as patients who died or required respiratory support (including CPAP, BiPAP, intubation, or high-flow nasal cannula). **Controls:** 1,383,241 individuals enrolled in the cohorts and not included as cases.

Summary statistics of thyroid dysfunctions were from The ThyroidOmics Consortium (https://transfer.sysepi.medizin.uni-greifswald.de/thyroidomics/datasets/). The GWAS summary comprised 19 independent cohorts (19). The hypothyroidism cohort included 3,340 cases with thyroid-stimulating hormone (TSH) levels above the reference range, and the hyperthyroidism cohort included 1,840 cases with TSH levels below the cohort-specific reference range. In addition, 49,983 control individuals with TSH levels within the reference range were included in the cohort. Patients receiving medication for thyroiditis were excluded. Genotype data were estimated from the 1,000 genomes, Project Phase 1, Version 3 ALL Population Reference Panel; and all analyses were adjusted for age, age squared, and sex. Details of the 19 cohorts can be found in the original article (19).

The ethical approval and consent information for the above summary statistics were taken from the original publication.

### Selection of genetic instruments

Single nucleotide polymorphisms (SNPs) that met the p < 5 × 10^−8^ threshold and minor allele frequency >1% were included to avoid potential statistical bias from the original GWAS. We then retained only those SNPs with linkage disequilibrium (R2 < 0.01) clustered in genomic regions 5,000 kbp apart. We used the PhenoScanner database (http://www.phenoscanner.medschl.cam.ac.uk/phenoscanner) to examine the selected instruments variables associated with other phenotypes that may at risk of affecting outcome. rs597808, selected SNP for exposure of hypothyroidism, was correlated significantly with hematological phenotypes (e.g., platelet count, total eosinophil basophil count, percentage of neutrophils in granulocytes, and lymphocyte count). Some studies have shown that changes in blood counts are a marker of SARS-CoV-2 infection and severity, with 25% of patients with COVID19 showing various forms of leucopenia (WBC < 4.00E+09 cells/liter) and the majority (63%) showing lymphocytopenia (20). One other study found that reduced platelet counts can lead to severe COVID-19 (21). Therefore, we conducted MR analysis before and after removed rs597808. We did not detect any SNP association with body mass index, smoking, or drinking, which may be at risk of affecting hyperthyroidism or hypothyroidism when the outcome of MR was thyroid dysfunction (22, 23).

R^2^ represents the ability of genetic variables to explain the exposures. In our study, the explained variances ranged from 7.1% to 17.6%. In addition, we used F statistics to avoid any weak instrumental variables (F > 10). The detailed information for the selected SNP is shown in the **Supplementary File**.

### Mendelian randomization analysis

**Figure 1:**
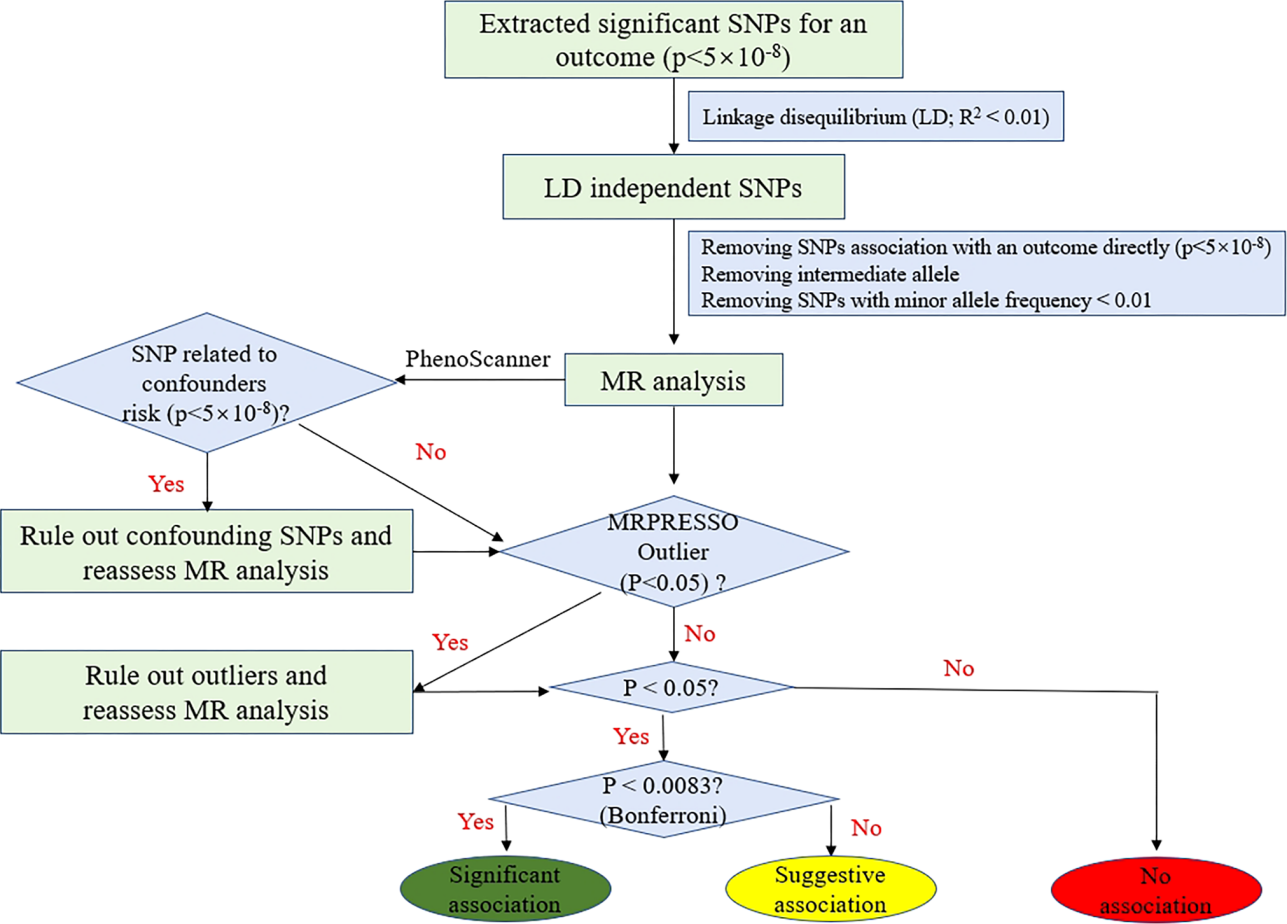
Flow chart about the analytical methods and how the MR analysis was performed step by step.

We performed a Wald ratio to assess the effect of exposure on an outcome for each genetic instrument. Then, we used the inverse variance-weighted (IVW) method by combining each effect size as the main analysis to estimate the causal effect of exposure on outcome in the fixed-effects model (24). Moreover, the MR-Egger and weighted-median (WM) methods were applied as supplements. The MR-Egger method was based on the Instrument Strength Independent of Direct Effect assumption. The MR-Egger method often takes inaccurate and statistically low results, especially in the case of the small size of SNPs (e.g., <10) (25). In addition, the value of the MR-Egger intercept term is far from zero, indicating horizontal pleiotropy (*P* < 0.05). Therefore, the MR-Egger method was mainly conducted to detect pleiotropy in our MR study. The WM analysis was more reliable if more than one-half of SNPs are invalid genetic instruments (e.g., due to pleiotropy) (26). We applied Cochrane’s Q-value to examine the heterogeneity, and we adopted the IVW random-effects method as the main effect size if heterogeneity existed. In addition, MR-PRESSO (27) was performed to detect any outlier which may lead to the heterogeneity. If we detected outliers, then they would be removed, and we reassessed the MR effect. We adjusted the multiple testing by a Bonferroni-corrected threshold of *p* < 0.0083 (p< 0.05/3/2). The *p*-values between 0.0083 and 0.05 were considered suggestive associations. A flow chart about how the MR analysis was performed step by step is shown in **Figure 1**.

We applied the “TwoSampleMR” and “MR-PRESSO” packages to our MR study; all statistical analyses were performed on the basis of the R software 4.1.1, and the data visualization was conducted in R software and STATA 12.0.

## Results

### Causal association of thyroid dysfunction with COVID-19 *via* forward MR

There was no evidence supporting hypothyroidism as a risk or protective factor for COVID-19 susceptibility, hospitalization, and very severe disease phenotype (IVW: OR = 0.971, 95% CI = 0.826–1.083, *P* = 0.193; OR = 0.983, 95% CI = 0.862–1.121, *P* = 0.798; and OR = 0.911, 95% CI = 0.746–1.112, *P* = 0.359; respectively). In addition, the association between hyperthyroidism and COVID-19 susceptibility, hospitalization, and very severe disease phenotype risk was not observed (IVW: OR = 0.983, 95% CI = 0.948–1.019, *P* = 0.705; OR= 0.938, 95%CI= 0.873-1.007, p=0.077; and OR = 0.921, 95% CI = 0.826–1.026, *P* = 0.136; respectively).

We detected a significant correlation between the genetic instrument rs597808 and hematological features of hyperthyroidism, which may be a confounding factor for the COVID-19 phenotype. We then removed rs597808 and reassessed the MR analysis but reached the same conclusions as the former. On the basis of Q-tests, MR-Egger intercepts, and MR-PRESSO to detect associations between thyroid dysfunction and COVID-19 risk, there was no significant heterogeneity, horizontal pleiotropy, or outliers. Detailed results of the different MR analyses are shown in **Table 2**.

**Table 2:** MR estimates for the causal effect of thyroid dysfunction on COVID-19.

	Outcome	NSNP	IVW		Weighted Median		MR-Egger		*P(I^2^)*	*P(_pleiotropy)_*	*P(_Global_)*
			OR (95% CI)	*P*	OR (95% CI)	*P*	OR (95% CI)	*P*			
**Hyper**	COVID-19 susceptibility	8	0.983 (0.948, 1.019)	0.705	0.972 (0.930, 1.016)	0.202	0.970 (0.835, 1.127)	0.705	0.847	0.864	0.835
	COVID-19 hospitalization	8	0.938 (0.873, 1.007)	0.077	0.936 (0.853, 1.028)	0.165	0.918 (0.684, 1.231)	0.752	0.619	0.587	0.619
	COVID-19 very severe	8	0.921 (0.826, 1.026)	0.136	0.947 (0.820, 1.094)	0.458	0.935 (0.587, 1.488)	0.786	0.448	0.950	0.473
**Hypo**	COVID-19 susceptibility	7	0.971 (0.826, 1.083)	0.193	0.959 (0.907, 1.015)	0.153	0.946 (0.826, 1.083)	0.458	0.711	0.701	0.730
	COVID-19 hospitalization	7	0.983 (0.862, 1.121)	0.798	0.903 (0.792, 1.028)	0.141	1.195 (0.791, 1.807)	0.436	0.037	0.373	0.053
	COVID-19 s very severe	7	0.911 (0.746, 1.112)	0.359	0.852 (0.689, 1.053)	0.138	0.943 (0.466, 1.908)	0.877	0.057	0.923	0.060
Hypo-excluded^*^	COVID-19 susceptibility	6	0.973 (0.928, 1.021)	0.267	0.956 (0.899, 1.016)	0.149	0.944 (0.825, 1.082)	0.456	0.593	0.667	0.646
	COVID-19 hospitalization	6	1.001 (0.871, 1.167)	0.914	0.995 (0.866, 1.143)	0.939	1.181 (0.759, 1.837)	0.503	0.043	0.497	0.063
	COVID-19 very severe	6	0.948 (0.765, 1.174)	0.627	0.971 (0.796, 1.185)	0.776	0.918 (0.446, 1.887)	0.827	0.061	0.930	0.116

Hypo-excluded^*^: MR analysis (exposure: hypothyroidism; outcome: COVID-19) after excluded rs597808 (PMID: 27863252), which significantly associated with hematological traits by performing PhenoScanner datasets; hematological parameters are markers of COVID-19 infection and severity; NSNP, number of single-nucleotide polymorphism; MR, Mendelian randomization; IVW, inverse variance weighting; OR, odds ratio. The I^2^ statistic was used to present the heterogeneity among estimates for each SNP in one analysis. P(_Global_): The p-value for the global test in the MR-PRESSO. P(_pleiotropy)_: The p-value for the intercept in the MR-Egger regression was used present the pleiotropy (p < 0.05). Hyper, hyperthyroidism; Hypo, hypothyroidism.

### Causal association of COVID-19 with thyroid dysfunction *via* reverse MR

**Figure 2:**
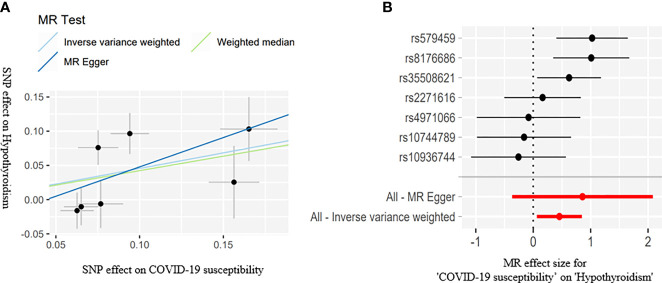
Scatter plots **(A)** for estimating causal effects of genetically predicted susceptibility on risk of hypothyroidism. Each black point representing the effect sizes of each SNP on the exposure (horizontal axis) and on the outcome (vertical axis) is plotted with error bars corresponding to each standard error (SE). The slope of each line corresponds to the combined estimate using each method of the IVW (light blue line), the MR-Egger regression (blue line), and the weighted median (light green line). Forest plots **(B)** of susceptibility on the risk of hypothyroidism; the red points showed the combined causal estimate using all SNPs together in a single instrument, using two different methods (MR-Egger and IVW).

**Figure 3:**
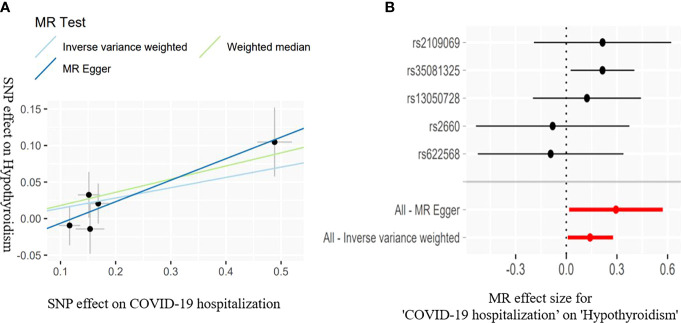
Scatter plots **(A)** for estimating causal effects of genetically predicted susceptibility on risk of hypothyroidism. Each black point representing the effect sizes of each SNP on the exposure (horizontal axis) and on the outcome (vertical axis) is plotted with error bars corresponding to each standard error (SE). The slope of each line corresponds to the combined estimate using each method of the IVW (light blue line), the MR-Egger regression (blue line), and the weighted median (light green line). Forest plots **(B)** of susceptibility on the risk of hypothyroidism; the red points showed the combined causal estimate using all SNPs together in a single instrument, using two different methods (MR-Egger and IVW).

The IVW estimate suggested that the susceptibility of COVID-19 and its severity may increase the risk of hypothyroidism. In addition, we deem that the association was suggestive because the p-values were between 0.0083 and 0.05 (Bonferroni-corrected threshold P = 0.0083). The MR results are shown in **Table 3** and **Figures 2**, **3**.

**Table 3:** MR estimates for the causal effect of COVID-19 on thyroid dysfunction.

	Outcome	NSNP	IVW		Weighted Median		MR-Egger		*P(I^2^)*	*P(_pleiotropy)_*	*P(_Global_)*
			OR (95% CI)	*P*	OR (95% CI)	*P*	OR (95% CI)	*P*			
**COVID-19** **susceptibility**	Hyper	7	0.963 (0.589, 1.575)	0.881	1.072 (0.667, 1.725)	0.773	2.745 (0.825, 9.132)	0.161	0.027	0.128	NA
	Hypo	7	1.577 (1.065, 2.333)	**0.022**	1.527 (1.042, 2.240)	**0.029**	2.358 (0.682, 8.032)	0.228	0.037	0.525	0.062
**COVID-19** **hospitalization**	Hyper	5	1.131 (0.936, 1.367)	0.203	1.200 (0.980, 1.469)	0.077	1.221 (0.889, 1.670)	0.214	0.284	0.185	0.324
	Hypo	5	1.151 (1.004, 1.319)	**0.042**	1.197 (1.023, 1.401)	**0.023**	1.342 (1.017, 1.771)	**0.038**	0.599	0.302	0.574
**COVID-19** **very severe**	Hyper	8	1.069 (0.959, 1.193)	0.228	1.094 (0.948, 1.263)	0.220	1.238 (0.955, 1.607)	0.158	0.612	0.269	0.532
	Hypo	8	1.103 (0.963, 1.263)	0.158	1.133 (1.006, 1.277)	**0.039**	1.334 (0.979, 1.818)	0.118	0.019	0.234	0.049
**COVID-19** **very severe outlier^#^**	Hypo	7	1.060 (0.954, 1.177)	0.276	1.126 (1.002, 1.262)	**0.043**	1.182 (0.920, 1.528)	0.191	0.245	0.106	0.251

Severity outlier^#^: MR analysis was reassessed (exposure: very severe COVID-19; outcome: hypothyroidism) after removed the MRPRESSO outlier (rs111837807; outlier test, p = 0.008); NSNP, number of single-nucleotide polymorphism; MR, Mendelian randomization; IVW, inverse variance weighting; OR, odds ratio. The I^2^ statistic was used to present the heterogeneity among estimates for each SNP in one analysis. P(_Global_): The p-value for the global test in the MR-PRESSO. P(_pleiotropy)_: The p-value for the intercept in the MR-Egger regression was used present the pleiotropy (p < 0.05). Hyper, hyperthyroidism; Hypo, hypothyroidism. Bold values indicate p<0.05.

COVID-19 susceptibility (IVW: OR = 1.577, 95% CI = 1.065–2.333, *P* = 0.022; WM: OR = 1.527, 95% CI = 1.042–2.240, *P* = 0.029) and hospitalization (IVW: OR = 1.151, 95% CI = 1.004–1.319, *P* = 0.042; WM: OR = 1.197, 95% CI = 1.023-1.401, *P* = 0.023) have a suggestive association with hypothyroidism. There was no association between the severe disease phenotype of COVID-19 and hypothyroidism. The light heterogeneity was excited when the susceptibility and the severe disease phenotype of COVID-19 were used as the exposure, so we performed the IVW random-effects method as the main analysis. The directional pleiotropy was not detected by the MR-Egger intercept. An outlier was detected by the MR-PRESSO test when the severe disease phenotype was used as the exposure, and we reassessed the MR after removing the outlier (**Table 3**).

Any type of COVID-19 phenotypes was not the risk or protective factor for hyperthyroidism (P > 0.05) in our study. The light heterogeneity was excited when the susceptibility phenotype of COVID-19 was used as the exposure, so we used the IVW random-effects method for our MR analysis. The directional pleiotropy and outliers were not detected by the MR-Egger intercept and MR-PRESSO, respectively.

The detailed MR results and sensitivity analysis for the previous results are presented in **Table 3**.

## Discussions

Our bidirectional two-sample MR study included two independent consortiums, and only European ancestry was retained. All genetic instruments were rigorously screened by conducting the PhenoScanner. Finally, our MR studies inferred suggestive causal effects of COVID-19 susceptibility and its severity on a higher risk of hypothyroidism.

Some previous observational studies have provided evidence of COVID-19–related primary hypothyroidism (8, 28, 29). Several studies have suggested that hypothyroidism may be caused by direct damage caused by SARS-CoV-2. Angiotensin-converting enzyme 2 (ACE2) and transmembrane protease serine 2 (TMPRSS2) binding is a key complex in SARS-CoV-2–infected hosts (30, 31). Notably, ACE2 and TMPRSS2 are expressed at high levels in the thyroid gland, even higher than in the lung tissue (32). Tee et al. (29) reported a case of overt primary hypothyroidism induced by autoimmune thyroiditis a week after mild COVID-19 symptoms were resolved. A case of hypothyroidism due to Hashimoto’s thyroiditis in a 45-year-old man was described (29). A study described that about 7% of patients who survived from severe COVID-19 might have a risk of persistent hypothyroidism, mostly due to Hashimoto’s thyroiditis (33). Another study reported that an 18-year-old woman who was hospitalized with severe viral thyroiditis had a positive oropharyngeal swab for SARS-CoV-2 15 days earlier and had mild symptoms of COVID-19 (34). In addition, some other studies reported that subacute viral thyroiditis appears 16–36 days after symptoms of COVID-19 disappear (35, 36). It is worth noting that the original GWAS defined hyperthyroidism and hypothyroidism based on TSH levels, and some patients with subclinical hypothyroidism might be included in the GWAS. There are some observational studies regarding subclinical hypothyroidism after COVID-19 infection. A clinical study by Burekovic et al. (37) showed that, at an average of 2 months after COVID-19 infection, some patients developed subclinical hypothyroidism, and another study (38) showed that seven of the 71 patients infected with COVID-19 included developed subclinical hypothyroidism.

Hypothyroidism is a broad spectrum of disorders that may be related to autoimmune disease or other processes such as viral thyroiditis. COVID-19 is associated with some autoimmune diseases other than thyroid disorder, which have been described in many studies, such as Guillain–Barre syndrome, autoimmune hemolytic anemia, and systemic lupus erythematosus (39). Antibodies against SARS-CoV-2 can react with various organs, including the thyroid gland, which can cause damage (40). ACE-2, the receptor through which SARS-COV-2 infects the host cells, is expressed by various tissues, including the thyroid. Therefore, the SARS-COV-2 virus will be present not only in the lung tissue but also in the thyroid cells. Hence, the patients may develop subacute thyroiditis following SARS-CoV-2 infection (41). Our MR study was limited by the original GWAS datasets, which were unable to distinguish between these different types of thyroid dysfunction, which may be the main source of heterogeneity. However, combining the results of this study with previous observational studies, we deem that COVID-19 susceptibility and its severity on the higher risk of any type of hypothyroidism.

At present, WHO has already made elderly, pregnant women, and people with heart failure, uncontrolled diabetes, and cancer-related complications as susceptible people of COVID-19. However, such general guidelines do not provide information about the COVID-19 risk of patients with thyroid problems. Most (42–46) epidemiological studies deem that thyroid dysfunction did not have higher rates of COVID-19 susceptibility and its severity, which is consistent with our conclusion. In an epidemiological study in Europe, patients with hyperthyroidism and hypothyroidism did not have an increased risk of COVID-19 infection (45). Two retrospective studies in the US (10) and Iran (46) show that patients with COVID-19 with hypothyroidism will not increase the rates of hospitalization or mortality. There may be multiple impacts of thyroid dysfunction on the risk of COVID-19 susceptibility and its severity due to mixed confounding factors such as an antithyroid drug. Boelaert et al. (47) reported that antithyroid drug-induced neutropenia, but not thyroid disorder status, may promote the progression of COVID-19 and its symptoms (47). We think that whether thyroid dysfunction increases the risk of COVID-19 requires further study, which can provide guidance on the management of thyroid disorder during the COVID-19 pandemic. We note that a recent MR study on COVID-19 and thyroid function showed that patients with COVID-19 susceptibility but not severe were more likely to develop hypothyroidism (48). We point out that the reason our study also found a potential correlation between patients with severe COVID-19 and hypothyroidism is that we included a larger number of patients with COVID-19 GWAS compared to study by Li et al. In addition, our inclusion criteria for genetic instruments were more stringent, as we excluded any genetic instruments that could be associated with confounding factors, whereas the study by Li et al. did not, thus giving our study more credibility.

Our study used the MR method by performing genetic instruments to examine the causal relationship between the characteristics of thyroid dysfunction and COVID-19 risk, and MR analysis can eliminate confounding factors as much as possible. In addition, a large size of independent datasets was used to obtain genetic instruments, which can mitigate the risk of bias in our results due to a few cases. However, there are some limitations to our study. This study included heterogenous in datasets, and the original GWAS datasets are unable to distinguish between these different types of thyroid dysfunction. However, combined with previous epidemiological studies, we point out that COVID-19 is associated with various types of hypothyroidism; thus, further research is required. As a two-sample MR analysis requires that both samples come from the same population, our study includes only participants of European ancestry; therefore, the generalization of these results to other populations requires further study.

## Conclusion

Our results identified that COVID-19 susceptibility and its severity might increase the risk of hypothyroidism. Therefore, patients infected with SARS-CoV-2 should strengthen the monitoring of thyroid function. However, the effect of thyroid dysfunction on COVID-19 susceptibility and its severity was not found in this MR study.

## Data availability statement

The original contributions presented in the study are included in the article/**Supplementary Material**. Further inquiries can be directed to the corresponding author.

## Ethics statement

This study only used published or publicly available data. Ethical approval for each study included in the investigation can be found in the original publications (including informed consent from each participant).

## Author contributions

ZZ and TF designed the study. ZZ and TF conducted data analysis. ZZ conceived the project and wrote the manuscript. YL revised and approved the paper. All authors contributed to the article and approved the submitted version.

## Funding

This work was supported by the Scientific Research Project of Xi'an [No. 2019115213YX007SF040 (2)], the Scientific Talents type II Project of Xi'an (No. J201902045),the Shaanxi Provincial Health Research Fund Project (No. 2022B003) and Xi’an No.3 Hospital (No. Y20201001). Those funders had no role in the study design, data collection and analysis, decision to publish, or preparation of the manuscript.

## Acknowledgments

We gratefully thank The Thyroid Omics Consortium and COVID19-hg Consortium for providing statistical data.

## Conflict of interest

The authors declare that the research was conducted in the absence of any commercial or financial relationships that could be construed as a potential conflict of interest.

## Publisher’s note

All claims expressed in this article are solely those of the authors and do not necessarily represent those of their affiliated organizations, or those of the publisher, the editors and the reviewers. Any product that may be evaluated in this article, or claim that may be made by its manufacturer, is not guaranteed or endorsed by the publisher.

## References

[1] Wiersinga WJ Rhodes A Cheng AC Peacock SJ Prescott HC. Pathophysiology, transmission, diagnosis, and treatment of coronavirus disease 2019 (Covid-19): A review. Jama (2020) 324(8):782–93. doi: 10.1001/jama.2020.12839 32648899

[2] Aung N Khanji MY Munroe PB Petersen SE. Causal inference for genetic obesity, cardiometabolic profile and covid-19 susceptibility: A mendelian randomization study. Front Genet (2020) 11:586308. doi: 10.3389/fgene.2020.586308 33262790 PMC7686798

[3] Stefan N Birkenfeld AL Schulze MB Ludwig DS. Obesity and impaired metabolic health in patients with covid-19. Nat Rev Endocrinol (2020) 16(7):341–2. doi: 10.1038/s41574-020-0364-6 PMC7187148 32327737

[4] Elrayess MA Cyprian FS Abdallah AM Emara MM Diboun I Anwardeen N. Metabolic signatures of type 2 diabetes mellitus and hypertension in covid-19 patients with different disease severity. Front Med (2021) 8:788687. doi: 10.3389/fmed.2021.788687 PMC8784560 35083246

[5] Trump S Lukassen S Anker MS Chua RL Liebig J Thürmann L. Hypertension delays viral clearance and exacerbates airway hyperinflammation in patients with covid-19. Nat Biotechnol (2021) 39(6):705–16. doi: 10.1038/s41587-020-00796-1 33361824

[6] Damara FA Muchamad GR Ikhsani R Hendro Syafiyah AH Bashari MH. Thyroid disease and hypothyroidism are associated with poor covid-19 outcomes: A systematic review, meta-analysis, and meta-regression. Diabetes Metab syndr (2021) 15(6):102312. doi: 10.1016/j.dsx.2021.102312 34731819 PMC8530797

[7] Hariyanto TI Kurniawan A. Thyroid disease is associated with severe coronavirus disease 2019 (Covid-19) infection. Diabetes Metab syndr (2020) 14(5):1429–30. doi: 10.1016/j.dsx.2020.07.044 PMC7387272 32755846

[8] Lania A Sandri MT Cellini M Mirani M Lavezzi E Mazziotti G. Thyrotoxicosis in patients with covid-19: The thyrcov study. Eur J Endocrinol (2020) 183(4):381–7. doi: 10.1530/eje-20-0335 PMC9494315 32698147

[9] Trimboli P Camponovo C Scappaticcio L Bellastella G Piccardo A Rotondi M. Thyroid sequelae of covid-19: A systematic review of reviews. Rev endocr Metab Disord (2021) 22(2):485–91. doi: 10.1007/s11154-021-09653-1 PMC8038866 33843008

[10] van Gerwen M Alsen M Little C Barlow J Naymagon L Tremblay D. Outcomes of patients with hypothyroidism and covid-19: A retrospective cohort study. Front Endocrinol (2020) 11:565. doi: 10.3389/fendo.2020.00565 PMC7461836 33013686

[11] Duntas LH Jonklaas J. Covid-19 and thyroid diseases: A bidirectional impact. J Endocr Soc (2021) 5(8):bvab076. doi: 10.1210/jendso/bvab076 34189381 PMC8135350

[12] Scappaticcio L Pitoia F Esposito K Piccardo A Trimboli P. Impact of covid-19 on the thyroid gland: An update. Rev endocr Metab Disord (2021) 22(4):803–15. doi: 10.1007/s11154-020-09615-z PMC7688298 33241508

[13] Chen M Zhou W Xu W. Thyroid function analysis in 50 patients with covid-19: A retrospective study. Thyroid: Off J Am Thyroid Assoc (2021) 31(1):8–11. doi: 10.1089/thy.2020.0363 32600165

[14] Lisco G De Tullio A Jirillo E Giagulli VA De Pergola G Guastamacchia E. Thyroid and covid-19: A review on pathophysiological, clinical and organizational aspects. J endocrinol Invest (2021) 44(9):1801–14. doi: 10.1007/s40618-021-01554-z PMC7992516 33765288

[15] Smith GD Ebrahim S. ‘Mendelian randomization’: Can genetic epidemiology contribute to understanding environmental determinants of disease? Int J Epidemiol (2003) 32(1):1–22. doi: 10.1093/ije/dyg070 12689998

[16] Davies NM Holmes MV Davey Smith G. Reading mendelian randomisation studies: A guide, glossary, and checklist for clinicians. BMJ (Clinical Res ed) (2018) 362:k601. doi: 10.1136/bmj.k601 PMC6041728 30002074

[17] Davey Smith G Hemani G. Mendelian randomization: Genetic anchors for causal inference in epidemiological studies. Hum Mol Genet (2014) 23(R1):R89–98. doi: 10.1093/hmg/ddu328 PMC4170722 25064373

[18] The covid-19 host genetics initiative, a global initiative to elucidate the role of host genetic factors in susceptibility and severity of the sars-Cov-2 virus pandemic. Eur J Hum genet: EJHG (2020) 28(6):715–8. doi: 10.1038/s41431-020-0636-6 PMC7220587 32404885

[19] Teumer A Chaker L Groeneweg S Li Y Di Munno C Barbieri C. Genome-wide analyses identify a role for Slc17a4 and aadat in thyroid hormone regulation. Nat Commun (2018) 9(1):4455. doi: 10.1038/s41467-018-06356-1 30367059 PMC6203810

[20] Huang C Wang Y Li X Ren L Zhao J Hu Y. Clinical features of patients infected with 2019 novel coronavirus in wuhan, China. Lancet (London England) (2020) 395(10223):497–506. doi: 10.1016/s0140-6736(20)30183-5 PMC7159299 31986264

[21] Zhou F Yu T Du R Fan G Liu Y Liu Z. Clinical course and risk factors for mortality of adult inpatients with covid-19 in wuhan, China: A retrospective cohort study. Lancet (London England) (2020) 395(10229):1054–62. doi: 10.1016/s0140-6736(20)30566-3 PMC7270627 32171076

[22] Andersen SL Olsen J Wu CS Laurberg P. Smoking reduces the risk of hypothyroidism and increases the risk of hyperthyroidism: Evidence from 450,842 mothers giving birth in Denmark. Clin Endocrinol (2014) 80(2):307–14. doi: 10.1111/cen.12279 23808881

[23] Chen X Wang JJ Yu L Wang HY Sun H. The association between bmi, smoking, drinking and thyroid disease: A cross-sectional study in wuhan, China. BMC endocr Disord (2021) 21(1):184. doi: 10.1186/s12902-021-00852-0 34517857 PMC8436425

[24] Burgess S Butterworth A Thompson SG. Mendelian randomization analysis with multiple genetic variants using summarized data. Genet Epidemiol (2013) 37(7):658–65. doi: 10.1002/gepi.21758 PMC4377079 24114802

[25] Bowden J Davey Smith G Burgess S. Mendelian randomization with invalid instruments: Effect estimation and bias detection through egger regression. Int J Epidemiol (2015) 44(2):512–25. doi: 10.1093/ije/dyv080 PMC4469799 26050253

[26] Bowden J Davey Smith G Haycock PC Burgess S. Consistent estimation in mendelian randomization with some invalid instruments using a weighted median estimator. Genet Epidemiol (2016) 40(4):304–14. doi: 10.1002/gepi.21965 PMC4849733 27061298

[27] Verbanck M Chen CY Neale B Do R. Detection of widespread horizontal pleiotropy in causal relationships inferred from mendelian randomization between complex traits and diseases. Nat Genet (2018) 50(5):693–8. doi: 10.1038/s41588-018-0099-7 PMC6083837 29686387

[28] Muller I Cannavaro D Dazzi D Covelli D Mantovani G Muscatello A. Sars-Cov-2-Related atypical thyroiditis. Lancet Diabetes Endocrinol (2020) 8(9):739–41. doi: 10.1016/s2213-8587(20)30266-7 PMC7392564 32738929

[29] Tee LY Harjanto S Rosario BH. Covid-19 complicated by hashimoto’s thyroiditis. Singapore Med J (2021) 62(5):265. doi: 10.11622/smedj.2020106 32668831 PMC8801861

[30] Lam SD Bordin N Waman VP Scholes HM Ashford P Sen N. Sars-Cov-2 spike protein predicted to form complexes with host receptor protein orthologues from a broad range of mammals. Sci Rep (2020) 10(1):16471. doi: 10.1038/s41598-020-71936-5 33020502 PMC7536205

[31] Li MY Li L Zhang Y Wang XS. Expression of the sars-Cov-2 cell receptor gene Ace2 in a wide variety of human tissues. Infect Dis poverty (2020) 9(1):45. doi: 10.1186/s40249-020-00662-x 32345362 PMC7186534

[32] Coperchini F Ricci G Croce L Denegri M Ruggiero R Villani L. Modulation of ace-2 mrna by inflammatory cytokines in human thyroid cells: A pilot study. Endocrine (2021) 74(3):638–45. doi: 10.1007/s12020-021-02807-w PMC8256224 34224085

[33] Caron P. Thyroid disorders and sars-Cov-2 infection: From pathophysiological mechanism to patient management. Annales d’endocrinol (2020) 81(5):507–10. doi: 10.1016/j.ando.2020.09.001 PMC7498405 32950466

[34] Brancatella A Ricci D Viola N Sgrò D Santini F Latrofa F. Subacute thyroiditis after sars-Cov-2 infection. J Clin Endocrinol Metab (2020) 105(7):1–4. doi: 10.1210/clinem/dgaa276 PMC7314004 32436948

[35] Brancatella A Ricci D Cappellani D Viola N Sgrò D Santini F. Is subacute thyroiditis an underestimated manifestation of sars-Cov-2 infection? Insights from a case series. J Clin Endocrinol Metab (2020) 105(10):dgaa537. doi: 10.1210/clinem/dgaa537 32780854 PMC7454668

[36] Chakraborty U Ghosh S Chandra A Ray AK. Subacute thyroiditis as a presenting manifestation of covid-19: A report of an exceedingly rare clinical entity. BMJ Case Rep (2020) 13(12):e239953. doi: 10.1136/bcr-2020-239953 PMC10577769 33370933

[37] Burekovic A Halilovic D Sahbaz A. Hypothyroidism and subclinical hypothyroidism as a consequence of covid-19 infection. Med Arch (Sarajevo Bosnia Herzegovina) (2022) 76(1):12–6. doi: 10.5455/medarh.2022.76.12-16 PMC8976890 35422565

[38] Zhang Y Lin F Tu W Zhang J Choudhry AA Ahmed O. Thyroid dysfunction may be associated with poor outcomes in patients with covid-19. Mol Cell Endocrinol (2021) 521:111097. doi: 10.1016/j.mce.2020.111097 33278491 PMC7709789

[39] Vojdani A Vojdani E Kharrazian D. Reaction of human monoclonal antibodies to sars-Cov-2 proteins with tissue antigens: Implications for autoimmune diseases. Front Immunol (2020) 11:617089. doi: 10.3389/fimmu.2020.617089 33584709 PMC7873987

[40] Tutal E Ozaras R Leblebicioglu H. Systematic review of covid-19 and autoimmune thyroiditis. Travel Med Infect Dis (2022) 47:102314. doi: 10.1016/j.tmaid.2022.102314 35307540 PMC8930178

[41] Chen W Tian Y Li Z Zhu J Wei T Lei J. Potential interaction between sars-Cov-2 and thyroid: A review. Endocrinology (2021) 162(3):1–13. doi: 10.1210/endocr/bqab004 PMC7953946 33543236

[42] Bhatraju PK Ghassemieh BJ Nichols M Kim R Jerome KR Nalla AK. Covid-19 in critically ill patients in the Seattle region - case series. New Engl J Med (2020) 382(21):2012–22. doi: 10.1056/NEJMoa2004500 PMC7143164 32227758

[43] Dworakowska D Grossman AB. Thyroid disease in the time of covid-19. Endocrine (2020) 68(3):471–4. doi: 10.1007/s12020-020-02364-8 PMC7275975 32507963

[44] Kim SY Yoo DM Min CY Choi HG. The effects of previous thyroid disease on the susceptibility to, morbidity of, and mortality due to covid-19: A nationwide cohort study in south Korea. J Clin Med (2021) 10(16):3522. doi: 10.3390/jcm10163522 34441818 PMC8396860

[45] Brix TH Hegedüs L Hallas J Lund LC. Risk and course of sars-Cov-2 infection in patients treated for hypothyroidism and hyperthyroidism. Lancet Diabetes Endocrinol (2021) 9(4):197–9. doi: 10.1016/s2213-8587(21)00028-0 PMC7906640 33617779

[46] Daraei M Hasibi M Abdollahi H Mirabdolhagh Hazaveh M Zebaradst J Hajinoori M. Possible role of hypothyroidism in the prognosis of covid-19. Internal Med J (2020) 50(11):1410–2. doi: 10.1111/imj.15000 PMC7753499 33215834

[47] Boelaert K Visser WE Taylor PN Moran C Léger J Persani L. Endocrinology in the time of covid-19: Management of hyperthyroidism and hypothyroidism. Eur J Endocrinol (2020) 183(1):G33–g9. doi: 10.1530/eje-20-0445 PMC7938012 32438340

[48] Li GH Tang CM Cheung CL. Covid-19 and thyroid function: A bi-directional two-sample mendelian randomization study. Thyroid: Off J Am Thyroid Assoc (2022). doi: 10.1089/thy.2022.0243 35734897

