# Supplementary figures and images for: Causal associations between thyroid dysfunction and COVID-19 susceptibility and severity: A bidirectional Mendelian randomization study
Source: Front Endocrinol (Lausanne). 2022 Sep 6;13:961717. doi: 10.3389/fendo.2022.961717 (PMC9485491; doi:10.3389/fendo.2022.961717)

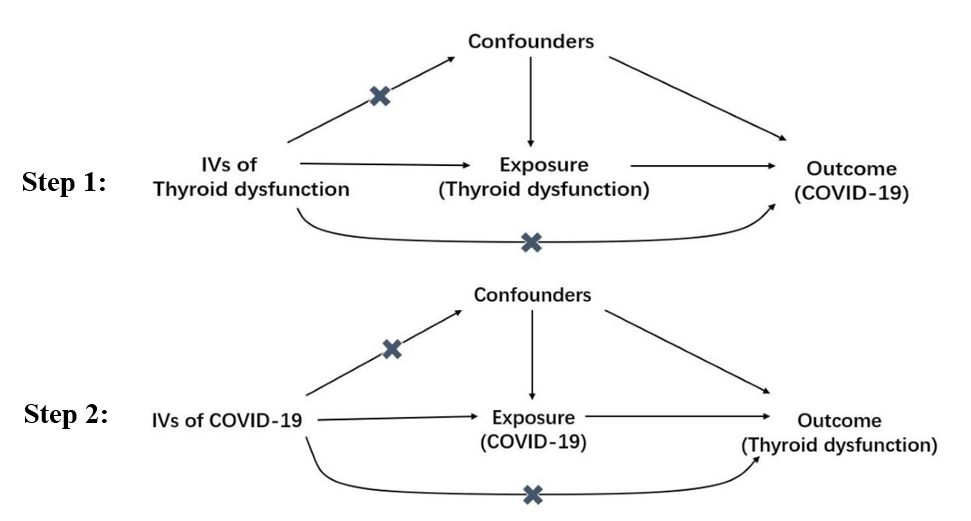

Supplement: Supplementary file 1 [file Image_1.jpeg]
